# Identification of dental calcification stages as a predictor of skeletal development phase

**DOI:** 10.1590/2177-6709.26.4.e2119292.oar

**Published:** 2021-09-10

**Authors:** Patrícia Ravena M. REBOUÇAS, Catarina Ribeiro Barros de ALENCAR, Maria Jacinta A. L. L. A. ARRUDA, Rosa Helena W. LACERDA, Daniela P. de MELO, Ítalo de M. BERNARDINO, Patrícia M. BENTO

**Affiliations:** 1 Universidade Estadual da Paraíba, Departamento de Odontologia (Campina Grande/PB, Brazil).; 2 Universidade Federal de Campina Grande (Campina Grande/PB, Brazil).

**Keywords:** Carpal bones, Cervical vertebrae, Orthodontics, Radiography

## Abstract

**Objective:**

This study aimed to establish a correlation between the stages of tooth calcification of mandibular canines and second molars with the phases of skeletal development.

**Methods:**

In a consecutive series of panoramic, cephalometric and hand-wrist radiographs of 113 individuals (60 females and 53 males) with an average age of 12.24 ± 1.81 years, the stages of mandibular canine and second molar calcification, cervical vertebrae maturation indicators (CVMI) and skeletal maturity indicators (SMI) were classified. The variables were correlated by means of the Spearman’s Rank test: chronological age, SMI, CVMI and tooth calcification stages. In order to assess whether the CVMI and tooth calcification stages were significant predictors of the SMI, an ordinal regression analysis was carried out.

**Results:**

The stages of CVMI (OR = 16.92; CI 95% = 6.45-44.39; *p*< 0.001) and calcification of the second molars (OR = 3.22; CI 95% = 1.50-6.92; *p*= 0.003) were significant predictors of SMI, however similar result was not observed for canines (OR = 0.52, CI 95% = 0.18-1.54; *p*= 0.239). Calcification stage E for boys, and E and F for girls corresponded to the pre-peak phase of pubertal growth. Stages G and H for boys, and F and G for girls coincided with peak of growth. In the final growth phase, the majority of second molars presented with root apex closure (stage H).

**Conclusion:**

The stages of calcification of the second molar may be considered predictors of the stage of skeletal development in the population studied.

## RESUMO

**Objetivo:**

O presente estudo objetivou estabelecer uma correlação entre os estágios de calcificação dentária de caninos e segundos molares inferiores e as fases do desenvolvimento esquelético.

**Métodos:**

Em uma série consecutiva de radiografias panorâmicas, cefalométricas e de mão e punho de 113 indivíduos (60 meninas e 53 meninos) com idade média de 12,24 ± 1,81 anos, foram classificados os estágios de calcificação do canino e do segundo molar inferiores, indicadores de maturação das vértebras cervicais (IMVC) e indicadores de maturação esquelética (IME). As variáveis foram correlacionadas pelo teste de Correlação de Rank de Spearman: idade cronológica, IME, IMVC e estágios de calcificação dentária. A fim de avaliar se os estágios do IMVC e da calcificação dentária foram preditores significativos do IME, foi realizada uma análise de regressão ordinal.

**Resultados:**

Os estágios de IMVC (OR = 16,92; IC 95% = 6,45-44,39; *p*< 0,001) e calcificação dos segundos molares (OR = 3,22; IC 95% = 1,50-6,92; *p*= 0,003) foram preditores significativos de IME; no entanto, esse não foi o caso com dentes caninos (OR = 0,52, IC 95% = 0,18-1,54; *p*= 0,239). Os estágios de calcificação E para meninos e E e F para meninas corresponderam à fase pré-pico de crescimento puberal. Os estágios G e H para meninos e F e G para meninas coincidiram com o pico de crescimento. Na fase final de crescimento, a maioria dos segundos molares apresentou fechamento do ápice radicular (estágio H).

**Conclusão:**

Os estágios de calcificação do segundo molar podem ser considerados preditores do estágio de desenvolvimento esquelético na população estudada.

## INTRODUCTION

The correction of malocclusion in patients with skeletal discrepancies and the prognosis for orthodontic treatment are heavily influenced by growth.^1^ ^,^ ^2^ ^,^ ^3^ Skeletal development is responsible for guiding clinical decisions on the use of extraoral traction and functional appliances, the need for tooth extraction and referral for orthognathic surgery.^4^ Therefore, identification of the different phases of growth is a crucial aspect of orthodontic planning.^2^

Skeletal development evaluation using hand-wrist radiographs is traditionally regarded as the gold standard;^5^ however, there is a concern over the additional exposure to radiation resulting from the use of this method.^6^ More recently, changes in the size and shape of cervical vertebrae has received growing interest as a biological indicator of skeletal development.^2^ ^,^ ^7^ However, the reproducibility of this method has been called into question^6^ ^,^ ^8^ and requires observer experience to assess the growth events.^9^

In this context, dental development should be studied in parallel with other indicators of physiological growth.^1^ ^,^ ^10^ ^,^ ^11^ Tooth development can be evaluated through the dental eruption phase or tooth calcification stage. It has been reported that calcification stage is considered more reliable.^12^ Associations between the stages of tooth calcification and indicators of skeletal development probably allow the clinicians to identify more easily the phases of pubertal growth using panoramic radiography.^11^ ^,^ ^13^ ^,^ ^14^

Considering the ethnic variations that influence skeletal development, environmental conditions and regional/climatic variations,^3^ ^,^ ^15^ the methods used to evaluate skeletal development and tooth calcification must be tested on different populations.^16^ No prior studies have investigated the applicability of the methods in children and adolescents from the northeastern region of Brazil. Moreover, previous studies focused on isolated comparisons between the hand-wrist maturation methods and tooth calcification stages^4^ ^,^ ^11^ ^,^ ^16^ or tooth development and cervical vertebrae maturation.^17^ ^-^ ^20^ Given the above, the aim of this study was to assess the correlation between the stages of tooth calcification and skeletal development in a group of Brazilian children and adolescents. The hypothesis of the study was that the cervical vertebrae maturation indicators (CVMI) and tooth calcification stages could be used as skeletal maturity indicators (SMI) in Brazilian children.

## MATERIAL AND METHODS

### POPULATION AND SAMPLE

The research protocol performed in this study was approved by the Research Ethics Committee from *Universidade Estadual da Paraíba* (CAAE: 24266713.6.0000.5187) and followed the guidelines of the Declaration of Helsinki. The research consisted of an analytical-observational and retrospective protocol. The study comprised of a series of panoramic, cephalometric and hand-wrist radiographs from children and adolescents of the Northeastern region of Brazil. The radiographs were taken for orthodontic purposes, and patients medical records were selected consecutively, thus reducing sampling bias. The individuals who had a prior history of orthodontic treatment, systemic diseases, endocrine/nutritional changes, trauma in the region of cervical vertebrae, hand-wrist or face and developmental anomalies in the teeth were excluded from the study. Patients with radiographic images conducted on different dates were also not eligible to participate. Radiographs of 113 individuals (60 females and 53 males) were included in the sample, with an average age of 12.24 ± 1.81, ranging from 9 to 15.5 years old.

### ACQUISITION AND ANALYSIS OF THE RADIOGRAPHS

The digital images were obtained using the Gendex^®^ Orthoralix 9200 (Gendex Dental Systems, Milan, Italy), and analyzed using the software Foton X (CDT Software, Bauru, Brazil) in a dark room with the aid of a 15-in video monitor (Dell Computer Corp., Round Rock, USA). Image manipulation tools were used to adjust brightness and contrast, and to provide a proportional zoom of 150%. The Kappa coefficient was used to assess intra and inter-rater reproducibility. Agreement between the two measurement times (two evaluations were carried out in the pilot phase, with an 10-day interval) was considered excellent (k = 0.99). Inter-observer agreement was also excellent (k = 0.81). The radiographic images were evaluated blinded and separately by two examiners. In the event of a disagreement between the rates, a third examiner was consulted, to establish the stage of development.

### EVALUATION OF THE STAGES OF TOOTH CALCIFICATION

The Demirjian method^21^ classifies tooth development into eight stages (Table 1). The mandibular canine and second molar on the left side of each panoramic radiograph were evaluated. In the eventuality that the left-side mandibular tooth was missing, the corresponding right-side tooth was examined.

**Table 1:** Tooth calcification stages according to Demirjian et al.^21^ method.

STAGE	EVENTS
A	Calcification of single occlusal points, without fusion of different calcifications.
B	Fusion of mineralization points; the contour of the occlusal surface is recognizable.
C	Enamel formation has been completed at the occlusal surface, and dentin formation has commenced. The pulp chamber is curved, and no pulp horns are visible.
D	Crown formation has been completed to the level of the cementoenamel junction. Root formation has commenced. The pulp horns are beginning to differentiate, but the walls of the pulp chamber remain curved.
E	The root length remains shorter than the crown height. The walls of the pulp chamber are straight, and the pulp horns have become more differentiated than in the previous stage. In molars the radicular bifurcation has begin to calcify.
F	The walls of the pulp chamber now form an isosceles triangle, and the root length is equal to or greater than the crown height. In molars, bifurcation has developed sufficiently to give the roots a distinct form.
G	The walls of the root canal are now parallel, but the apical end is partially open. In molars, only the distal root is rated.
H	The root apex is completely closed (distal root in molars). The periodontal membrane surrounding the root and apex is uniform in width throughout.

### EVALUATION OF CERVICAL VERTEBRAE DEVELOPMENT

The cervical vertebrae maturation indicators (CVMI) proposed by Hassel and Farman^22^ and modified by Baccetti et al.^7^ consist of observation of anatomic changes in the second, third and fourth cervical vertebrae, examined on cephalometric radiographs. The subjects were grouped according to the growth phase,^18^ and the bone development events categorized from stages 1 to 6 (Table 2).

**Table 2:** Cervical vertebrae maturation indicators proposed by Baccetti et al.,^7^ and pubertal growth stages according Perinetti et al.^18^

STAGE	OSSIFICATION EVENTS	PUBERTAL GROWTH STAGE
1	The lower borders of all the three vertebrae (C2-C4) are flat. The bodies of both C3 and C4 are trapezoid in shape.	Onset
2	A concavity is present at the lower border of C2. The bodies of both C3 and C4 are still trapezoid in shape.	
3	Concavities at the lower borders of both C2 and C3 are present. The bodies of C3 and C4 may be either trapezoid or rectangular horizontal in shape.	Peak
4	Concavities at the lower borders of C2, C3, and C4 now are present. The bodies of both C3 and C4 are rectangular horizontal in shape.	
5	The concavities at the lower borders of C2, C3, and C4 are still present. At least one of the bodies of C3 and C4 is squared in shape. If not squared, the body of the other cervical vertebra is still rectangular horizontal.	End
6	The concavities at the lower borders of C2, C3, and C4 are still evident. At least one of the bodies of C3 and C4 is rectangular vertical in shape. If not rectangular vertical, the body of the other cervical vertebra is squared.	

### EVALUATION OF HAND-WRIST DEVELOPMENT

The skeletal maturity indicators (SMI) proposed by Fishman^23^ use four stages of bone development in six anatomic regions located on the thumb, third and fifth fingers, and the radius bone. The bone development events were categorized from stages 1 to 11 (Table 3). The growth phases were categorized in accordance with Gilsanz and Ratib,^24^ but the “late puberty” and “post-puberty” phases, corresponding to the final period of growth,^22^ were deemed to be the “post-puberty” phase, in order to permit equivalence of comparison with the method employed to assess vertebral development. 

**Table 3:** Skeletal maturity indicators proposed by Fishman,^23^ and pubertal growth stages suggested by Gilsanz and Ratib^24^.

STAGE	OSSIFICATION EVENTS	PUBERTAL GROWTH STAGE
1	The proximal phalanx of third finger shows equal widths of epiphysis and diaphysis.	Pre-puberty
2	The middle phalanx of third finger shows equal widths of epiphysis and diaphysis.	
3	The middle phalanx of fifth finger shows equal widths of epiphysis and diaphysis	
4	Appearance of adductor sesamoid of the thumb.	Puberty
5	Capping of epiphysis of distal phalanx on the third finger	
6	Capping of epiphysis on middle phalanx on the third finger	
7	Capping of epiphysis of middle phalanx on fifth finger.	
8	Fusion between epiphysis and diaphysis of the distal phalanx on the third finger.	Late puberty
9	Fusion between epiphysis and diaphysis of the proximal phalanx of the third finger.	
10	Fusion between epiphysis and diaphysis of middle phalanx on the third finger.	
11	Fusion of epiphysis and diaphysis seen in the radius.	Post-puberty

### STATISTICAL ANALYSIS

The data were analyzed using the IBM SPSS^®^ (IBM Corp. Chicago, Illinois, USA) version 20.0. The level of significance was set at 5%. Initially, a descriptive statistical analysis was carried out in order to characterize the sample. Then the nonparametric and/or ordinal variables chronological age, SMI, CVMI and mandibular canine and second molar calcification (in the non-categorized forms) were correlated through Spearman’s Rank Correlation test. The average chronological age for both sexes in the three phases of growth were compared by means of the Kruskal-Wallis test with a subsequent multiple comparison of means, adjusted using the Bonferroni method. Ordinal regression analysis was employed to investigate whether the CVMI stages and tooth calcification were significant predictors of the pubertal growth phase of SMI. The logistic model for ordinal responses has a simple interpretation and higher power.^25^ ^,^ ^11^ The Polytomous Universal Model (PLUM) was applied, incorporating the ordinal nature of the dependent variable into the analysis. Therefore, a logistic regression model was built with proportional-odds and logit function. The tests that evaluate goodness of fit and homogeneity of regression slopes were also conducted in order to analyze the validity of the models constructed.^26^

## RESULTS

The results of the Spearman correlation analysis between chronological age, SMI, CVMI and mandibular canine and second molar calcification are presented in Table 4. All the correlation coefficients were positive and significant (*p*< 0.01) for the whole sample and for males and females separately.

**Table 4:** Spearman’s Rank Correlation between chronological age, SMI, CVMI and stages of calcification of mandibular canines and second molars.

		CA	SMI	CVMI	CCS	MCS
TOTAL SAMPLE	CA	1				
	SMI	0.743*	1			
	CVMI	0.771*	0.903*	1		
	CCS	0.789*	0.712*	0.717*	1	
	MCS	0.833*	0.735*	0.662*	0.784*	1
BOYS	CA	1				
	SMI	0.856*	1			
	CVMI	0.886*	0.888*	1		
	CCS	0.789*	0.719*	0.748*	1	
	MCS	0.852*	0.803*	0.735*	0.812*	1
GIRLS	CA	1				
	SMI	0.858*	1			
	CVMI	0.847*	0.896*	1		
	CCS	0.810*	0.801*	0.756*	1	
	MCS	0.827*	0.805*	0.672*	0.760*	1

*Correlation significant at the 0.01 level (2-tailed); CA = Chronological age; SMI= Skeletal maturity indicators; CVMI = Cervical vertebrae maturation indicators; CCS = Canine calcification stages; MCS = Molar calcification stages.

Table 5 displays the distribution of chronological age of the subjects, according to sex and pubertal growth phase of SMI. Significant differences between the sexes and between phases of pubertal growth (*p*< 0.05) were observed. The peak and post-peak growth phases were attained earlier among girls (*p*< 0.05).

**Table 5:** Distribution of chronological ages for all subjects, grouped by sex and skeletal maturity indicators.

Pubertal growth stage	Chronological age (years)	
	Male	Female
	Mean (SD)	Mean (SD)
Onset	10.67 (1.20)^Aa^	10.15 (0.78)^Aa^
Peak	12.91 (1.09)^Ab^	10.74 (0.92)^Ba^
End	14.03 (1.03)^Ab^	13.23 (1.36)^Bb^

SD = Standard Deviation; Different letters (uppercase in the horizontal and lowercase in the vertical) indicate statistically significant differences (*p* < 0.05), according to the Bonferroni test.

The results of the ordinal regression analysis for the whole sample are displayed in Table 6. The analysis revealed that CVMI and the stages of second molar calcification were statistically significant predictors of SMI. Similar results were observed after performing ordinal regression analysis for the group of boys (Table 7) and girls (Table 8).

**Table 6:** Results of ordinal multinomial logistic regression analysis for total sample.

Predictors	Estimate	SE	Wald	OR	CI 95%	p-value
CVMI	2.83	0.49	33.04	16.92	6.45-44.39	< 0.001
CCS	-0.65	0.55	1.38	0.52	0.18-1.54	0.239
MCS	1.17	0.39	8.96	3.22	1.50-6.92	0.003

OR = odds ratio; IC = confidence interval; SE = standard error; CVMI = Cervical vertebrae maturation indicators; CCS = canine calcification stages; MCS = Molar calcification stages; Model Fitting Information (-2 log-likelihood intercept only = 205.490; -2 log-likelihood intercept and covariates = 48.208); Pseudo R-Square (Cox and Snell = 0.751; Nagelkerke = 0.854; McFadden = 0.657).

**Table 7:** Results of ordinal multinomial logistic regression analysis for boys.

Predictors	Estimate	SE	Wald	OR	CI 95%	p-value
CVMI	2.22	0.58	14.43	9.22	2.93-28.98	< 0.001
CCS	-0.43	0.80	0.28	0.65	0.14-3.14	0.594
MCS	1.63	0.72	5.15	5.13	1.25-21.06	0.023

OR = odds ratio; IC = confidence interval; SE = standard error; CVMI = Cervical vertebral maturation indicators; CCS = Canine calcification stages; MCS = Molar calcification stages; Model Fitting Information (-2 log-likelihood intercept only = 97.481; -2 log-likelihood intercept and covariates = 25.568); Pseudo R-Square (Cox and Snell = 0.743; Nagelkerke = 0.838; McFadden = 0.625).

**Table 8:** Results of ordinal multinomial logistic regression analysis for girls.

Predictors	Estimate	SE	Wald	OR	CI 95%	p-value
CVMI	3.51	0.94	14.04	33.34	5.33-208.66	< 0.001
CCS	-0.91	0.85	1.14	0.40	0.08-2.14	0.286
MCS	1.19	0.56	4.55	3.30	1.10-9.88	0.033

OR = odds ratio; IC = confidence interval; SE = standard error; CVMI = Cervical vertebral maturation indicators; CCS = Canine calcification stages; MCS = Molar calcification stages; Model Fitting Information (-2 log-likelihood intercept only = 105.856; -2 log-likelihood intercept and covariates = 26.938); Pseudo R-Square (Cox and Snell = 0.732; Nagelkerke = 0.860; McFadden = 0.691).

The distribution of the stages of tooth calcification and vertebral development by sex and phases of pubertal growth defined by SMI can be seen in Table 9. In the pre-peak period, a considerable distribution of the stages of tooth calcification was found for both sexes. However, considering the second molar as the more reliable tooth for identifying skeletal development in this sample, it was noted that calcification stage E, for boys, and stages E and F for girls were most common in this period of growth. As far as vertebral development is concerned, stage 2 was the most frequent among boys, while stage 3 was the most frequent among girls.

**Table 9:** Distribution of calcification stages of teeth and CVMI, according to sex and SMI.

Variable		Sex		
		Male	Female	Total
		n (%)	n (%)	n (%)
ONSET	Canine calcification stages			
	D	0 (0.0)	0 (0.0)	0 (0.0)
	E	0 (0.0)	1 (11.1)	1 (3.3)
	F	12 (57.1)	5 (55.6)	17 (56.7)
	G	5 (23.8)	2 (22.2)	7 (23.3)
	H	4 (19.0)	1 (11.1)	5 (16.7)
	Molar calcification stages			
	D	1 (4.8)	1 (11.1)	2 (6.7)
	E	10 (47.6)	3 (33.3)	13 (43.3)
	F	4 (19.0)	3 (33.3)	7 (23.3)
	G	4 (19.0)	2 (22.2)	6 (20.0)
	H	2 (9.5)	0 (0.0)	2 (6.7)
	Cervical vertebrae maturation indicators			
	1	5 (23.8)	3 (33.3)	8 (26.7)
	2	10 (47.6)	2 (22.2)	12 (40.0)
	3	6 (28.6)	4 (44.4)	10 (33.3)
	4	0 (0.0)	0 (0.0)	0 (0.0)
	5	0 (0.0)	0 (0.0)	0 (0.0)
	6	0 (0.0)	0 (0.0)	0 (0.0)
PEAK	Canine calcification stages			
	D	0 (0.0)	0 (0.0)	0 (0.0)
	E	0 (0.0)	0 (0.0)	0 (0.0)
	F	1 (7.1)	3 (18.8)	4 (13.3)
	G	4 (28.6)	10 (62.5)	14 (46.7)
	H	9 (64.3)	3 (18.8)	12 (40.0)
	Molar calcification stages			
	D	0 (0.0)	1 (6.2)	1 (3.3)
	E	0 (0.0)	2 (12.5)	2 (6.7)
	F	1 (7.1)	7 (43.8)	8 (26.7)
	G	8 (57.1)	5 (31.2)	13 (43.3)
	H	5 (35.7)	1 (6.2)	6 (20.0)
	Cervical vertebrae maturation indicators			
	1	0 (0.0)	0 (0.0)	0 (0.0)
	2	1 (7.1)	0 (0.0)	1 (3.3)
	3	5 (35.7)	5 (31.2)	10 (33.3)
	4	6 (42.9)	9 (56.2)	15 (50.0)
	5	2 (14.3)	2 (12.5)	4 (13.3)
	6	0 (0.0)	0 (0.0)	0 (0.0)
END	Canine calcification stages			
	D	0 (0.0)	0 (0.0)	0 (0.0)
	E	0 (0.0)	0 (0.0)	0 (0.0)
	F	0 (0.0)	1 (2.9)	1 (1.9)
	G	1 (5.6)	3 (8.6)	4 (7.5)
	H	17 (94,4)	31 (88.6)	48 (90.6)
	Molar calcification stages			
	D	0 (0.0)	1 (2.9)	1 (1.9)
	E	0 (0.0)	0 (0.0)	0 (0.0)
	F	0 (0.0)	1 (2.9)	1 (1.9)
	G	3 (16.7)	9 (25.7)	12 (22.6)
	H	15 (83.3)	24 (68.6)	39 (73.6)
	Cervical vertebrae maturation indicators			
	1	0 (0.0)	0 (0.0)	0 (0.0)
	2	0 (0.0)	0 (0.0)	0 (0.0)
	3	1 (5.6)	0 (0.0)	1 (1.9)
	4	3 (16.7)	4 (11.4)	7 (13.2)
	5	4 (22.2)	8 (22.9)	12 (22.6)
	6	10 (55.6)	23 (65.7)	33 (62.3)

Bold values indicate the highest frequencies in the categories of the most reliable predictors of skeletal maturation.

In the peak growth phase, the majority of second molars were identified in stage G or H among the boys, while for the girls, stages F or G were the most frequent. With regard to cervical vertebrae development, stage 4 predominated among both boys and girls.

In the post-peak period, the second molar in the majority of boys and girls was observed in stage H. Stage 6, attributed to CVMI in the final phase of growth, was the most frequent among both boys and girls. Generally, the stages of tooth calcification in boys were found to be more advanced when compared to girls in the same period of skeletal development.

## DISCUSSION

Many methods have been suggested for assessing pubertal growth in orthodontic diagnosis and planning. Evaluation of the stages of tooth calcification has the advantage of permitting easy assessment through radiographs present in routine orthodontic documentation.^17^ ^,^ ^10^

Some studies have shown significant correlation between the stages of tooth calcification and different indicators of skeletal development.^1^ ^,^ ^10^ ^,^ ^13^ ^,^ ^14^ ^,^ ^17^ ^,^ ^20^ On the other hand, some authors have reported lower or insignificant correlation between skeletal and dental development.^27^ ^,^ ^28^ ^,^ ^29^ The lack of agreement in previous studies is partially a result of ethnic differences^1^ ^,^ ^16^ and different methods used to evaluate skeletal development and tooth calcification.^17^

Hand-wrist radiography is the most common indicator used by orthodontists to evaluate skeletal development. According to the assumption of Todd,^30^ in a uniformly developed skeleton, any area would show the same state of development. More recently, Hoseini et al.^8^ suggested that, although biologically the skeletal development in both sexes has a closer correlation with pubertal growth spurts than chronological age, hand-wrist radiographs are not totally appropriate for this purpose, as bones undergo constant change during development. Hand-wrist radiographic images as a tiny part of this system, cannot be representative of the whole skeleton.

The present study aimed to evaluate the correlation between phases of skeletal development based on hand-wrist bones maturation^23^ and a cervical vertebrae development method, as a second reference for maturity,^7^ in respect of tooth calcification.^21^ The practical goal was to estimate the applicability of the use of panoramic radiographs as a clinically useful resource for identifying the phases of pubertal growth.

Through the findings of this study, the methods used to evaluate hand-wrist and cervical vertebrae development were found to be equivalent for the whole sample and separately by sex. Moreover, the appearance of the events of skeletal development was seen to occur earlier in girls than in boys, in agreement with previous findings on different populations.^1^ ^,^ ^13^ ^,^ ^14^ ^,^ ^29^

In the present sample, the Spearman correlation between the SMI and stages of canine and second molar calcification were significant and high for boys (r = 0.719 and 0.803) and girls (r= 0.801 and 0.805). The same was found between CVMI and tooth calcification, though with slightly lower correlation values for the second molar in boys (r = 0.735) and girls (r = 0.672) and the canines in girls (r = 0.756). However, Lopes et al^11^ asserted that a high correlation is a natural tendency, because both tooth calcification and skeletal development are events that are in progress in growing individuals. Thus, a high correlation coefficient does not furnish information on whether the stage of tooth calcification is satisfactory for identifying the stage of skeletal development.^10^

For this reason, an ordinal regression analysis was performed, which showed that only the second molar was considered a significant predictor of skeletal development. Therefore, it is important to highlight that previous studies recommending the use of stages of calcification of mandibular canines as indicators of pubertal growth were limited to an analysis of correlation.^4^ ^,^ ^14^

Many authors^10^ ^,^ ^13^ ^,^ ^14^ ^,^ ^15^ ^,^ ^17^ have also found that calcification stages of the mandibular second molar showed greater correlation with skeletal development than other teeth, as it tends to take longer to develop to a more advanced age, normally presenting apex closure at the age of 16 years.^12^ ^,^ ^17^

For the second molars evaluated in this study, it was noted that the calcification stage E for boys and stages E and F for girls corresponded to the period preceding the peak of pubertal growth, estimated by means of SMI method. In these children, stage 2 of the CVMI was most frequent among boys, and stage 3 among girls. Kumar et al.^17^ found that for both sexes, stage E of the mandibular second molar was the most common in the stage 2 of the CVMI. In Brazilian children,^11^ stages E and F for boys and D and E for girls were correlated to the pre-peak growth phase, estimated with the hand-wrist method.

In the peak growth period, the majority of second molars were identified in stage G or H in boys and F or G in girls. In both sexes, there was a predominance of stage 4 in the CVMI. Stages 3 and 4 of CVMI also represented the peak of pubertal growth in the children studied by Giri et al.^15^ in Nepal; and for these children, stages F and G of mandibular second molar calcification for females and stage G for males were correlated to peak pubertal growth. According to Kumar et al.^17^, in this phase, stages F and G was most frequent in stages 3 and 4 of the CVMI, similar to what was previously reported by other authors.^14^

In the post-peak period of growth, the second molar of the majority of boys and girls was observed in stage H, as evidenced by Litsas et al.^20^ in Greek children. In the present study, stage 6 attributed to CVMI in the final phase of growth was the most frequent for both boys and girls. In the findings of Kumar et al.^17^, stage H was associated with stages 5 and 6 of the CVMI (end of the growth spurt). In the final phase of pubertal growth, the majority of teeth evaluated by Lopes et al^11^ had already achieved apical closure. In girls, however, the majority of second molars were still found in stage G, evidencing a more accelerated tooth calcification in boys. This observation was confirmed by the present study, and has also been previously described.^1^ ^,^ ^13^ ^,^ ^14^

Thus, the simplicity of the evaluation of some teeth development according to Demirjian stages^21^ and correlations previously found between the stages of tooth calcification and the phase of pubertal growth^15^ ^,^ ^17^ ^,^ ^20^ ^,^ ^11^ allow to regard tooth development as a tool for an initial assessment of the child’s skeletal development. Caution is recommended when interpreting the results of this study and other cross-sectional research, because of the limited evaluation of growth. While the hypothesis of this study was accepted, since CVMI and tooth calcification stages are considered significant predictors of the SMI in Brazilian children, it is important to emphasize that the clinical use of stages of tooth calcification to establish phases of skeletal development cannot be derived from the present study. Lastly, the results presented here are an original contribution to the discussion on correlation between different evaluation methods to estimate the skeletal development in individuals in growth phase with orthodontic needs.

## CONCLUSIONS

The stages of calcification of the left mandibular second molar were considered to be better predictors of the skeletal development than that of the mandibular canine, and may be used in a preliminary identification of the phases of pubertal growth in the population studied. In the eventuality that the left-side mandibular tooth is missing, the corresponding, right-side one is examined. The timing of peak growth identified by SMI coincided with the calcification stages G and H for boys and F and G for girls, and stage 4 of CVMI.
